# Comparative morpho‐functional analysis of the humerus and ulna in three Western European moles species of the genus *Talpa*, including the newly described *T. aquitania*

**DOI:** 10.1111/joa.13772

**Published:** 2022-09-26

**Authors:** Pauline Costes, Estelle Klein, Arnaud Delapré, Céline Houssin, Violaine Nicolas, Raphaël Cornette

**Affiliations:** ^1^ Institut de Systématique, Evolution, Biodiversité (ISYEB) UMR 7205, Muséum National d'Histoire Naturelle, CNRS, SU, EPHE, UA Paris France; ^2^ Mecanismes Adaptatifs et Évolution UMR 7179, CNRS Muséum National d'Histoire Naturelle Paris France

**Keywords:** 3D geometric morphometrics, morpho‐functional, phenotypic integration, *Talpa*

## Abstract

The forelimb is involved in many behaviours including locomotion. Notably, the humero‐ulnar articulation, implicated in the elbow joint, is of particular importance for both mobility and stability. Functional constraints, induced in part by environmental plasticity, are thought to drive an important part of the bone shape as bone directly responds and remodels in response to both muscle and external forces. In this context, the study of subterranean moles is of particular interest. These moles occupy a hard and heavy medium in comparison with air or water, requiring a powerful body structure to shear and shift the soil. Their general morphology is therefore adapted to digging and to their subterranean lifestyle. The various morpho‐functional patterns, which drive diverse abilities according to the environment, are likely targets of natural selection and it is, therefore, useful to understand the relationships between the bone shape and their function. Here, we quantify, through 3D geometric morphometric methods, the interspecific variability in the morphology of the ulna and humerus of three *Talpa* species, including the new species *Talpa aquitania*, to infer their potential consequence in species digging performance. We also quantify shape covariation and morphological integration between the humerus and the ulna to test whether these bones evolve as a uniform functional unit or as more or less independent modules. Our results show that interspecific anatomical differences in the humerus and ulna exist among the three species. Shape changes are mostly located at the level of joints and muscle attachments. As the species tend to live in allopatry and the fossorial lifestyle induces strong ecological constraints, interspecific variations could be explained by the properties of the environment in which they live, such as the compactness of the soil. Our results also show that the humerus and ulna are highly integrated. The covariation between the humerus and ulna in moles is dominated by variation in the attachment areas and particularly of the attachment areas of shoulder muscles concerning the humerus, which affect the mechanical force deployed during locomotion and digging. This study also highlights that in the new species, *T. aquitania*, variations in anatomical structure (general shape and joints) exist and are related to the locality of collect of the individuals.

## Graphical abstract

3D geometric morphometrics has been applied on the humerus and ulna of three European mole species: *T. occidentalis*, *T. europeae* and including the newly described *T. aquitania*. Specific shape differences have been found both on the humerus and ulna, in relation with muscle attachments and their digging abilities. Intraspecific shape variation has been highlighted among the new species *T. aquitania* specimens linked to the collected localities.

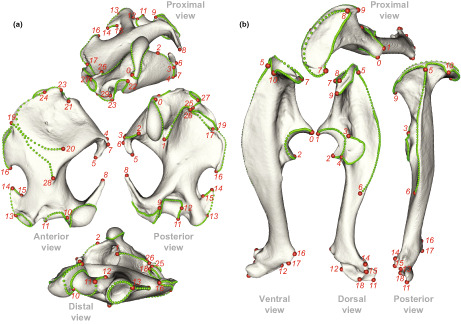

## 1 INTRODUCTION

Locomotion is crucial for an animal's ecology. Indeed, animals move in their home ranges to forage for food resources or pursue their prey, search for mating partners, avoid stressful environments or escape from potential predators (Biewener & Patek, 2003; Ewer, 1973; Martín‐Serra et al., 2014). During these behaviours, the limbs' role is to support the weight without breaking or collapsing and to resist the stresses and strains induced by locomotion and other behaviours. Specifically, it is the forelimb which is the principal support of body mass during locomotion in most quadrupedal mammals (Hanna et al., 2006; Lee et al., 1999; Raichlen et al., 2009; Reynolds, 1985; Schmitt & Lemelin, 2002; Walter & Carrier, 2007) but it is also involved in some other behaviours including social and feeding behaviours (Polly, 2007).

**FIGURE 1:**
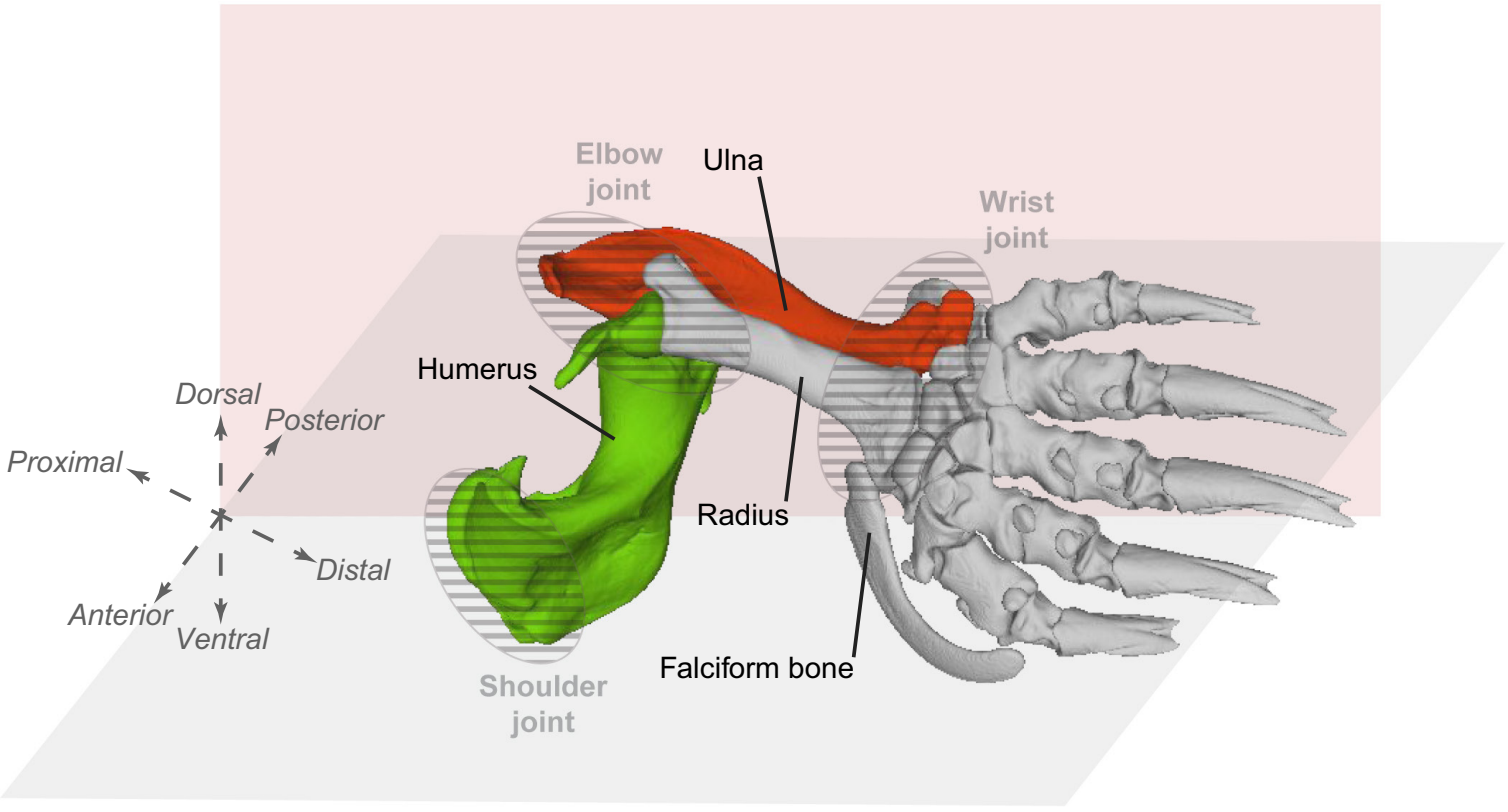
Illustration of the anatomy of the left forelimb of a mole specimen (*T. europaea*, specimen MNHN‐ZM‐MO‐1993‐3218). The humerus is coloured green and the ulna is coloured red.

The humerus, in the upper arm, and the ulna, associated with the radius in the lower arm, are two bones composing the forelimb (Figure 1). The humero‐ulnar articulation is implicated in the elbow joint, which is of particular importance to control both mobility and stability in quadrupedal animals (Fabre et al., 2014). The humerus of these animals is involved in the support of the anterior body and corresponds to the point of insertion for muscles moving the forelimb and the manus (Polly, 2007). It is also part of the shoulder joint, along with the scapula and clavicle. On the other hand, the ulna contributes to elbow joint stability and provides a point of insertion for elbow extensors (Polly, 2007). The distal end of the ulna is also part of the wrist joint.

The morphology of forelimb bones is shaped by functional, developmental and architectural constraints, as well as phylogeny (Cubo, 2004; Gould & Lewontin, 1979). Even if the shape of a bone results from numerous and complex processes, functional constraints, induced in part by environmental plasticity, are thought to drive an important part of the bone shape as bone directly responds and remodels in response to both muscle and external forces (Currey, 2002). For example, the size of muscle insertions, and the strength and curvature of bones are influenced by muscle volume and strength (Brassard et al., 2020; Cornette et al., 2015). These functional constraints can therefore impact the whole skeleton and induce a coordinated variation of bones. The tendency of morphological traits to varying in a coordinated manner is defined as morphological integration (Mitteroecker & Bookstein, 2007; Olson & Miller, 1951, 1958; Young & Hallgrímsson, 2005). In other cases, the constraints can have local effects or affect the skeletal parts differently. In this situation, the phenotypic covariation within parts (then referred to as a module) is greater than between the parts; this phenomenon is called modularity (Cornette et al., 2013; Mitteroecker & Bookstein, 2007). This can be due to the functional specialisation of a structure (Arias‐Martorell et al., 2014; Goswami et al., 2014; Young & Hallgrímsson, 2005). The various morpho‐functional patterns, which drive diverse abilities according to the environment, are likely targets of natural selection (Cornette et al., 2015). Understanding how bone shape is influenced and linking this morphological variation to its function will help us to understand evolutionary patterns.

In this context, the study of subterranean moles is of particular interest. These moles occupy a hard and heavy medium in comparison with air or water, requiring a powerful body structure to shear and shift the soil (Abe, 2001). Their general morphology is therefore adapted to digging and to their subterranean lifestyle, and several morphological, physiological and behavioural evolutionary convergences are observed amongst subterranean species of the Talpidae family (Sansalone et al., 2019). They share, for instance, a cylindrical body, a short neck, reduced eyes and external ears and ostensibly short limbs (Edwards, 1937; Nevo, 1979; Rohlf et al., 1996). The forelimb specialisation is the most noticeable (Figure 1). The forelimb is indeed the main locomotor structure used for digging. Thus, moles excavate their underground tunnel with broadened forefeet and strong claws (Motokawa, 2004). The manus has a special shape due to an increase in bone width and the development of an accessory bone, called the falciform bone (Edwards, 1937; Yalden, 1966). Bones are also modified and developed to allow the attachment of powerful muscles moving the forelimb (Edwards, 1937; Rose et al., 2013). Long bones (humerus, ulna and radius) are shorter but more robust than in other mammals (Edwards, 1937; Gambaryan et al., 2002; Hutchison, 1968; Piras et al., 2012).

The humerus of the fossorial moles (Scalopinae and Talpinae) has very distinct anatomy. Indeed, the bone appears very stocky, its width representing two‐thirds of its length (Campbell, 1939) with enlarged muscle attachment sites and pronounced joints compared to non‐burrowing sister taxa and other mammals. The two condyles are turned in opposite directions, resulting in a twist that is unique among mammals (Bickelmann et al., 2014). The 3D structure of the humerus is inscribed in a square whose diagonals correspond to axes of reinforcement of the architecture, determining either compact bone fields or vast indentations that protect the flexor muscles of the forearm (Castiella et al., 1992). Noticeable features are also present on the ulna. This bone exhibits, for instance, a greatly pronounced olecranon process with a crest expanding posteriorly (the posterior crest, after Hutchison, 1968) and a proximal crest expanding perpendicularly to the great axis of the bone, forming a large blade (Castiella et al., 1992; Edwards, 1937; Hutchison, 1968). As for the humerus, these prominent surfaces allow a greater attachment of muscles involved in forelimb movement. The ulna is interesting to study because it participates in the stabilisation of the elbow joint and is a point of insertion for elbow extensors (Polly, 2007). Geometric morphometric analyses on armadillo species have shown that adaptations to digging ability seem to be mostly related to a more secure elbow joint and more powerful muscles that control the forearm and hand (Milne et al., 2009). The ulna is one of the main bones undergoing stress during burrowing (Acuña et al., 2016). Moreover, digging ability has been previously shown to be well characterized by the relative length of the olecranon of the ulna, in other burrowing mammals such as armadillos (Vizcaíno et al., 1999). This olecranon has a very singular shape in moles (Castiella et al., 1992).

Species of the genus *Talpa* are subterranean and they are of particular interest due to a recent description of high cryptic species diversity within this genus (Bannikova et al., 2015; Demirtas et al., 2020; Kryštufek et al., 2018; Nicolas et al., 2017). Here, we focus on the newly described species *Talpa aquitania* Nicolas et al., 2016 and its two sister‐species, *Talpa europaea* (Linnaeus, 1758) and *Talpa occidentalis* (Cabrera, 1907). These three species co‐occur in south‐western Europe where they have allopatric or parapatric distributions: *T. occidentalis* is endemic to the Iberian Peninsula, *T. aquitania* is present in Northern Spain and south‐western France and *T. europaea* is widely distributed from north‐eastern France to Russia (Nicolas et al., 2017; Wilson & Mittermeier, 2018). *T. aquitania* and *T. occidentalis* co‐occur in Northern Spain. *T. aquitania* and *T. europaea* have mostly allopatric geographical distribution, even if small areas of contact between them are present in the Pyrénées mountain, the Var department and around the Loire river (Nicolas et al., 2021). The factors explaining their geographical distribution are still incompletely understood. Several phylogeographical studies highlighted the role of climatic changes in the Mio‐Pliocene as major forces driving extinction, diversification and migration in the genus *Talpa* and the role of Pleistocene climatic oscillations in causing range shrinkages and expansions that led to the current distribution of most *Talpa* species (Bannikova et al., 2015; Colangelo et al., 2010; Feuda et al., 2015; Loy et al., 2005; Nicolas et al., 2017). Several studies also showed interspecific competition as a driving force of mole distribution and coexistence: no more than two species (one larger, one smaller) occur in the same area. For example, sympatric pairs of congeneric moles occur in North America (genus *Scapanus* Pomel, 1848), the Mediterranean area (genus *Talpa*) and eastern Asia (genus *Mogera* Pomel, 1848) (Kryštufek & Motokawa, 2018; Loy et al., 2017). Species in such pairs differ in size, and the larger mole is normally more abundant and widespread and occupies a more extensive range of environmental conditions. It was shown that despite their widely corresponding distributions and their non‐overlapping sizes, the co‐occurring moles are only exceptionally syntopic, and can co‐occur only if they occupy different habitat patches in a habitat mosaic (Kryštufek & Motokawa, 2018). For example, in the Japanese moles, the dominant mole species, *Mogera wogura* (Temminck, 1842), is progressively expanding its range northwards, displacing the smaller species *M. imaizumii* (Kuroda, 1957) (Abe, 2001). Similarly, in the Balkans, *T. caeca* enclaves occurring in sympatry with *T. europaea* are displaced onto the drier, rockier area (Kryštufek, 1999). Soil hardness is an important factor affecting the geographical distribution of these species and it allows their coexistence only under very specific circumstances. Given the absence of syntopy between species and the limited availability of feeding resources underground, it was suggested that competitive interactions between moles are exerted via interference competition rather than via exploitative competition (Loy et al., 2017). As the forelimb forms a functional unit in most mammals, selection seems to operate more on the whole limb, in other words on the covariance between the structures that form it, rather than on each structure in isolation (Cheverud, 1982; Cornette et al., 2013). Selection pressures imposed by ecological specialisations are particularly important for the coordinated evolution of elements with the same function (Badyaev et al., 2005; Haber, 2014; McLean et al., 2018; Monteiro & Nogueira, 2010; Rossoni et al., 2019; Sherratt et al., 2017; Zeng, 1988). High integration maintains the relationships within functional units and appears essential for specialised lifestyles (Andjelković et al., 2017; Botton‐Divet et al., 2018; Martín‐Serra et al., 2015). Thus, functional specialisation in the forelimb in burrowing moles could induce strong integration within the limb (Sansalone et al., 2022; Young & Hallgrímsson, 2005).

This study has two aims. First, we will quantify, through 3D geometric morphometric methods, the interspecific variability in the morphology of the ulna and humerus of these three *Talpa* species to infer their potential consequences on species digging performance. Second, we will quantify shape covariation and morphological integration between the humerus and the ulna to test whether these bones evolve as a uniform functional unit or as more or less independent modules.

## 2 MATERIALS AND METHODS

### 2.1 Sample

Three *Talpa* sister‐species (Talpidae) found in Western Europe were studied: the European mole (*T. europaea)*, the Iberian mole (*T. occidentalis)* and the Aquitanian mole (*T. aquitania)* (Nicolas et al., 2017). We analysed 52 humeri and 52 ulnae (details in Table 1). All specimens used in this study come from the collections of the Muséum National d'Histoire Naturelle (Paris, France), for *T. aquitania* and *T. europaea*, or from the biological station of Donãna (Séville, Spain), for *T. occidentalis*. No specimen was trapped or euthanised for this project. Both sexes are represented in the sample and all individuals are considered fully adult, that is, specimens whose genitalia were well developed and whose weight corresponded to adult specimens. The thresholds used to define adult individuals are 65 g for males and 53 g for females of *T. occidentalis* (Barrionuevo et al., 2004), 87 g for males and 72 g for females of *T. europaea* (Crowcroft & Godfrey, 1960). There are no published data for *T. aquitania*, thus we took the same values as for *T. europaea*. 3D models of the humeri and ulnae were made by segmentation of CT scans with Avizo (Thermo Fischer Scientific, Waltham, MA). As far as possible, the left bones have been used. However, when the left humeri or ulnae were not well preserved, the right bones were symmetrized to obtain standardized data. A summary of the known information for each individual is available as a Table S1.

**TABLE 1:** Sample location and size for each species and sex.

Species	Female	Male	Indefinite	Location	Total
*T. europaea*	13	6	0	Côtes d'armor, Essonne, Ile‐et‐vilaine (France)	19
*T. occidentalis*	5	7	2	Lugo, Madrid (Spain)	14
*T. aquitania*	10	9	0	Aveyron, Gironde (France)	19
Total	28	22	2		52

### 2.2 Study of morphological variations using 3D geometric morphometrics

#### 2.2.1 Data acquisition and statistical analysis

**FIGURE 2:**
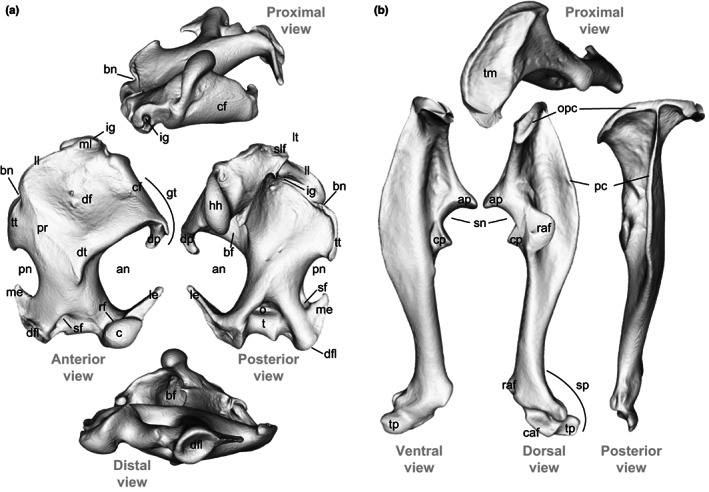
3D humerus (a) and ulna (b) of the species *Talpa aquitania*. Indications of the main anatomical features follow Bickelmann et al. (2014), Edwards (1937), Sánchez‐Villagra et al. (2004), Castiella et al. (1992) [humerus] and Hutchison (1968), Jullien (1967) and Whidden (2000) [ulna]. Humerus (a): an: anterior notch, bf: brachial fossa, bn: bicipital notch, c: capitulum, cf: clavicular facet, dfl: distal facet‐like process of the medial epicondyle, df: deltoid fossa, dp: deltoid process, dt: deltoid tuberosity, gt: greater tuberosity, hh: humeral head, ig: intertubercular (bicipital) groove, le: lateral epicondyle, ll: lateral lamina (bicipital ridge), lt: lesser tuberosity, me: medial epicondyle, ml: medial lamina on lesser tuberosity, o: olecranon fossa, pn: posterior notch, pr: pectoralis ridge, rf: radial fossa, sf: supracondylar foramen, slf: *subscapularis* ligament facet, t: trochlea, tt: teres tubercle ‐ Ulna (b): ap: anconeus process, caf: cuneiform articular facet, cp: coronoid process, opc: olecranon proximal crest, pc: posterior crest, raf: radial articular facet, sn: semilunar notch, sp: styloid process, tm: triceps area of insertion, tp: terminal process.

**FIGURE 3:**
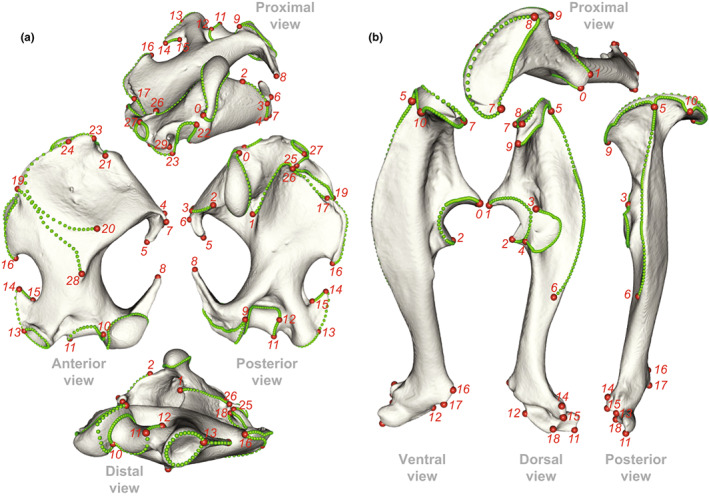
3D humerus (a) and ulna (b) of the species *Talpa aquitania*. Landmarks used in this study are illustrated by coloured spheres. Anatomical landmarks are in red and sliding semilandmarks are in green. We refer to the definitions of the landmarks available as a Table S2.

To analyse the shape variation of the humerus and ulna between the three *Talpa* species, we used geometric morphometrics. This method corresponds to the statistical analysis of landmark‐based shape variation (Zelditch et al., 2012). A set of 3D landmarks were manually placed on each 3D model, using the software Landmark (Landmark Editor Version 3.6: Institute for Data Analysis and Visualisation, Wiley et al., 2005). Two types of landmarks were chosen for analysis of the 52 humeri and ulnae of moles: anatomical landmarks which define fixed anatomical points on the bones and sliding semilandmarks which will enable the analysis of parts devoid of anatomical landmarks, such as the diaphyses and joint surfaces. Sliding semilandmarks were allowed to slide on 3D curves while minimising the bending energy between the template and the object to measure. The use of such landmarks is interesting because it allows the shape of the whole bone to be described and analysed, including areas without anatomical landmarks. (Gunz & Mitteroecker, 2013). We, thus, placed on the humerus 30 anatomical landmarks and 537 sliding semilandmarks to define the 62 3D curves (Figure 3a; Table S2a). Concerning the ulna, 19 anatomical landmarks and 360 sliding semilandmarks have been placed, to define the 39 3D curves (Figure 3b; Table S2b). Each anatomical landmark corresponds to the location of known anatomical features in moles of the genus *Talpa* (Figures 2, 3). The raw material (meshes and landmarks) is available on request.

Statistical analysis and 3D visualisation were performed using R software (version 4.1.1; R Development Core Team, 2021). A Generalized Procrustes analysis (GPA) by minimising Procrustes Shape Distances was performed on the data for both humeri and ulnae (Rohlf & Slice, 1990), using the “gpagen” function of the “geomorph” package (Adams et al., 2021). The GPA performs a rotation, translation and scaling of specimens (Gower, 1975; Rohlf & Slice, 1990). To visualise the interspecific and intraspecific shape differences in humerus and ulna, a principal component analysis (PCA) was first carried out on the superimposed landmarks coordinates, using the “gm.prcomp” function of the “geomorph” package (Adams et al., 2021). Then, multivariate analyses of variance (MANOVA) were used to statistically test the significance of the differences observed between the species, between locations in *T. aquitania* and between sexes. All the MANOVA tests were performed on more than 90% of the variance (the first 34 and 26 axes, respectively, for the humerus and ulna). Indeed, a dimensionality reduction was necessary to study these complex structures with large sets of 3D landmarks (Baylac & Frieß, 2005; Gunz & Mitteroecker, 2013).

#### 2.2.2 Analysis of size and allometry

Centroid size for a set of landmarks is the square root of the sums of squared distances between landmarks and their centroid and is used to represent the overall size of the landmark configuration. The effect of species on the centroid size of the humerus and ulna was assessed using an ANOVA and then pairwise species comparisons using *t*‐tests. Variation in size is an important determinant for variation in many other organismal traits (Klingenberg, 2016). The effect of allometry, defined as “size‐related changes in morphological traits” (Klingenberg, 2016), on the overall morphological change was therefore assessed. A Procrustes ANOVA (Goodall, 1991) was performed, using the “procD.lm” function in the “geomorph” package (Adams et al., 2021), to test the effect of allometry and assess whether it varies by species. The function quantifies the relative amount of shape variation attributable to the size and species of specimens, in a linear model and estimates the probability of this variation (“significance”), via distributions generated from resampling permutations. The function uses Procrustes distances among specimens, rather than explained covariance matrices among variables. With this approach, the sum‐of‐squared Procrustes distances are used as a measure of sums of squares (see Goodall, 1991). Using the “plotAllometry” function of the “geomorph” package (Adams et al., 2021), we found and visualised the major axis of covariation between centroid size (log10) and shape by a singular value decomposition of their cross products, a process known as two‐block partial least squares (PLS; Rohlf & Corti, 2000). This major axis of variation, called the common allometric component (CAC; Mitteroecker et al., 2004), approximates the mean direction of shape development. This approach, combining Procrustes ANOVA and visualisation using the CAC (e.g., Zhang & Schepartz, 2021), allows for good readability of the results. To obtain size‐corrected data, the residuals from the multivariate regression of shape on size were used, using the “vecx” function in the “Morpho” package (Schlager, 2017). They are in the same coordinate system as the original shape data, but the predicted component of shape variation has been removed (Klingenberg, 2016). For each bone, a PCA was performed on the allometry‐free residuals, using the “gm.prcomp” function in the “geomorph” package (Adams et al., 2021).

#### 2.2.3 Visualisation of shape variation

3D visualisations were generated, using the “Morpho” and “rgl” packages (respectively Schlager, 2017 and Murdoch & Adler, 2021), to compare shape variations associated with the minimum and maximum of the two first axes of the PCA. For the humerus, the fourth axis was also represented because it was the most representative of intraspecific variation in *T. aquitania*. As already shown by several authors (e.g., Fabre et al., 2015; Lefebvre et al., 2020; Mallet et al., 2019), the comparison of the shapes associated with the ends of each axis makes it possible to visualise the morphological differences, maximised by PCA, that exist between the three species. These figures were created after deformations of the most central specimens of the PCA (respectively, the humerus of *T. occidentalis* n°18,086–18,089 and ulna of *T. occidentalis* n°18,082) using the Thin‐Plate Spline (TPS) method and the “tps3d” function of the “Morpho” package (Schlager, 2017). These deformations are always carried out between only two items: a reference and a target. The TPS algorithm minimises the “bending energy” between two homologous points datasets, which means the energy of local deformation requires skipping from one shape to another (Gunz & Mitteroecker, 2013). Thus, the 3D humerus (n°18,086–18,089) and the 3D ulna (n°18,082) are deformed at first to match the consensus shape and a second time to match the theoretical shape associated with the maxima and minima of the axes. TPS, which is an interpolation technique, provides a one‐to‐one correspondence not only between the reference points of the starting shape and the target but also between each point of the 3D space in which the shapes associated with the minimum and maximum are embedded (Klingenberg, 2013). Then the shapes representing the minimum and maximum were compared using two visualisation methods. Both types of visualisation are based on direct superimposition of the entire bone surface using the “shade3d” function of the “rgl” package (Murdoch & Adler, 2021). In the first type, shape changes are visualised using a representation of the surface which is coloured according to a heat map representing the distance between the surfaces of the minimal and maximal shape to each point. “Cold” colours represent areas where the maximal shape recedes within the minimal shape and “warm” colours where the maximal shape bulges out of the minimal shape (Klingenberg, 2013). These figures, obtained using the “meshDist” function of the “Morpho” package (Schlager, 2017), show the overall changes in the shape of the bones and their intensity about each other (Figures 5 and 12b,c). In the second type of visualisation, the theoretical shapes associated with the minimum and maximum of the axes are both visible, in transparency, and in addition, in the manner of “lollipop” diagrams (Klingenberg, 2013), arrows indicate the displacement of the positions of the landmarks between these two shapes. These arrows start from the shape associated with the minimum of the axes and end at the shape associated with the maximum of the axes. The arrows are coloured according to a colour gradient, from light colours for the shortest arrows, associated with the smallest changes between the two shapes, to dark colours for the longest arrows, associated with the most significant changes. This technique allows the detail of changes in bone shape to be highlighted more accurately than the first method (Figures 7, 8, 9, 10, 11; Figure S3). We have chosen, for our visualisations, to use the least‐squares Procrustes superimposition (Dryden & Mardia, 2016; Goodall, 1991), which tends to spread the changes over several landmarks (Piras et al., 2020). However, there are other strategies for visualising a change in shape that are equivalent (Klingenberg, 2021). For example, the resistant‐fit superimposition tends to focus differences on one or a few landmarks (Chapman, 1990; Rohlf & Slice, 1990; Siegel & Benson, 1982). Therefore, it is important to keep in mind that there are many pairs of icons, which are equivalent in showing the same change in shape, but which result in different landmark shifts (Klingenberg, 2021). We used arrows (lollipop graphics), of varying intensity of colour, to highlight the areas where morphological differences are most important. From a biological point of view, shape changes cannot be attributed to individual landmarks (Pinocchio effect; Walker, 2000) but are inextricably associated with the space between them (Klingenberg, 2021). In addition to arrows, we have used 3D surfaces that allow for a more intuitive anatomical interpretation (Klingenberg, 2013). We are fully aware of the limitations of this type of visualisation.

### 2.3 Shape covariation between the humerus and ulna

To quantify and visualise shape covariation and the morphological integration between humerus and ulna, we performed two block PLS (Rohlf & Corti, 2000) implemented in the “two.b.pls” function of the “geomorph” package (Adams et al., 2021) for each dataset separately. This approach is relevant for understanding how correlation and independence patterns change within a species and evolve (Goswami & Polly, 2010). PLS has been used previously to compare the morphological integration of the limb long bone between different populations or species (e.g., in horses: Hanot et al., 2019 and in rhinoceroses: Mallet et al., 2020). This method allows us to extract the eigenvectors and eigenvalues from two blocks of covarying data, each of these blocks representing the shape variation of one bone, the ulna or the humerus. A covariance matrix is constructed by combining the two sets of variables, and PLS finds the principal components of covariation between these datasets. This method then generates the axes of variation common to both blocks, the first axis explaining most of the covariation, as in PCA (Goswami & Polly, 2010). Thus, patterns of covariance between the two blocks (humerus–ulna) can be explored, and the PLS axes can be plotted. Finally, a PLS coefficient (rPLS) is calculated allowing us to estimate the degree of morphological integration. A significance test is obtained by 10,000 permutations of the landmarks in one block relative to those in the other. A significant P‐value was obtained when the observed PLS was higher than those of a distribution of values obtained from randomly permuted blocks and implies that the blocks are significantly integrated. The percentage of total shape variance accounted for by PLS1 within each block was calculated. This is, within each module, the ratio between the variance of PLS1 scores and the sum of the variances of the shape coordinates of the landmarks in that module (Cardini, 2019).

## 3 RESULTS

### 3.1 Size and allometry

**FIGURE 4:**
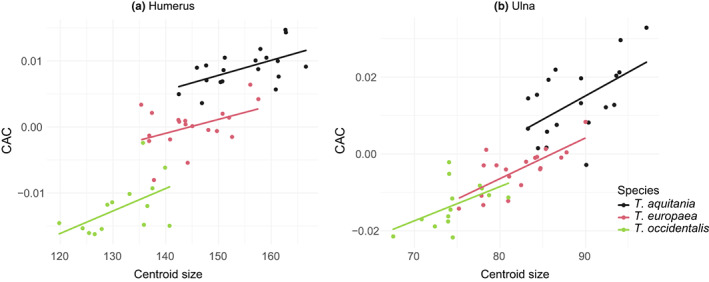
Multivariate regression with Procrustes coordinates (represented by CAC) and centroid size of the humerus (a) and ulna (b).

Species has an effect on the centroid size of the humerus and ulna (humerus: df = 2, F = 47.660, *P* = 3.210e‐12; ulna: df = 2, F = 53.360, *P* = 4.990e‐13). As can be seen in Figure 4 and Figure S1, *T. aquitania* has the largest humerus and ulna while *T. occidentalis* has the smallest. The size of the humerus and ulna of *T. europaea* is intermediate between the bones of *T. aquitania* and *T. occidentalis*. These size differences between the three species were confirmed by pairwise comparisons using a t‐test, available as a supplementary table (Table S4).

Allometry was tested in the humerus and ulna with multivariate regression using Procrustes coordinates and centroid size. The Procrustes ANOVA revealed significant overall allometry for the humerus (Table S3a, Rsq = 0.142, *P* = 0.001) and ulna (Table S3b, Rsq = 0.135, *P* = 0.001). This means that the morphological changes of the humerus and ulna are related to the size changes. However, no difference in static allometry is detected between the species for humerus (Table S3a, Rsq = 0.037, *P* = 0.249) and ulna (Table S3b, Rsq = 0.036, *P* = 0.244). As the slopes did not differ significantly between species, we were able to control for the effect of allometry and calculate ‘size‐corrected’ shapes (Viscosi & Cardini, 2011). PCA of the size‐corrected data (Figure S4) shows that there is a difference in bone shape between the three mole species that is not related to size variation. Indeed, this difference can be seen on the first axis for the humerus and the two first axes for the ulna. As our study questions the biomechanical constraints on the bones, linked to the biological reality under size selection, the size effect is retained in further analyses.

For both the humerus and ulna, there is variation in shape between large and small bones. The epicondyles are slightly longer in the smaller humeri than in the larger humeri. The pectoralis ridge and capitulum are more prominent in the smaller humeri. In contrast, the lateral lamina and teres tubercle are more developed in the large humeri. On the other hand, the posterior crest is more prominent in large ulnae. Finally, the large ulnae are more curved towards the dorsal part and the small ulnae are straighter. Detailed visualisations of morphological variations can be seen in Figure S3.

### 3.2 Morphological variations of the humerus and ulna

The PCA performed on the humerus (Figures 6a,b) and ulna (Figure 6c) highlights three distinct groups of individuals which correspond to the three species of moles. The MANOVA confirmed the existence of a significant difference in bone shape between the three mole species (humerus: F = 25.549, *P* < 2.200e‐16; ulna: F = 15.148, *P* < 2.200e‐16).

Concerning the humerus (Figures 6a,b), the first two axes of the PCA explain 20.72% of the total variance. The first axis of the PCA allows us to separate the specimens of *T. aquitania* and *T. occidentalis*, represented at the minimum end of the axis, and the specimens of *T. europaea*, represented at the maximum end of the axis. Indeed, the teres tubercle (Figure 7c) and the attachment facet of the subscapularis ligament (Figure 7d) are more developed in *T. occidentalis* and *T. aquitania*, than in *T. europaea*. The lateral epicondyle (Figures 7a,b) is more curved in *T. occidentalis* and *T. aquitania* than in *T. europaea*. Regarding joints, the capitulum (joint with the radius) is more bulging (Figure 7e) and the anterior aspect of the trochlea (joint with the ulna) (Figure 7f) is larger in *T. occidentalis* and *T. aquitania* than in *T. europaea*. In contrast, the pectoralis ridge (Figure 7i), the lateral lamina (Figure 7i) and lastly, the attachment area of the triceps brachii muscle (lateral and medial heads) (Figures 7g,h) are more developed in *T. europaea* than in the other two species.

The second axis of the PCA allows us to separate the specimens of *T. occidentalis* represented at the minimum end of the axis, and the specimens of *T. aquitania* represented at the maximum end of the axis. On this axis, *T. europaea* is in an intermediate position between the other two species. The deltoid tuberosity is more developed (Figures 8i,k) in *T. occidentalis* than in *T. aquitania*. The attachment areas of the ventral part of the costo‐scapularis and anterior long pectoralis muscles (Figures 8i,k), as well as the attachment facet of the subscapularis ligament (Figure 8d), are more developed in *T. occidentalis* than in *T. aquitania*. The distal facet‐like process of the medial epicondyle (Figure 8e) is also more developed in *T. occidentalis* than in *T. aquitania*. The medial epicondyle (Figure 8f) is longer in *T. occidentalis* than in *T. aquitania*, as well as the lateral epicondyle (Figure 8g). Regarding joints, the capitulum (joint with the radius) (Figure 8g) is larger and the olecranon fossa (Figure 8j) is deeper in *T. occidentalis* than in *T. aquitania*. In contrast, the pectoralis ridge, the lateral lamina (Figures 8i,k) and the teres tubercle (Figure 8h) are more developed in *T. aquitania* than in *T. occidentalis*.

The fourth axis (5.82% of the total variance) of the PCA allows the separation of the specimens of *T. aquitania* into two groups (Figure 6b). The MANOVA confirmed that humeri differ significantly between these two groups (F = 9.980, *P* = 0.019). These two groups correspond to the geographic area in which the specimens were collected. The maximum of axis 4 mainly explains the shape of the humerus of the specimens of *T. aquitania* that originated from Aveyron, and the minimum mainly explains the shape of the humerus of the specimens of *T. aquitania* that originated from Gironde. Indeed, the morphology corresponding to *T. aquitania* of Gironde and *T. aquitania* of Aveyron differ (Figure 9). The individuals living in Aveyron have larger and more robust humeri (Figures 9a,b), and the epicondyles are larger and longer (Figures 9c,d). The teres tubercle is also longer and more developed in Aveyron than in Gironde (Figure 9(f)). Lastly, the clavicular facet and humeral head are more developed in this locality (Figure 9g). In contrast, the capitulum is larger (Figure 9h), the posterior notch is smaller (Figure 9i) and the deltoid tuberosity is strengthened (Figure 9j) in Gironde than in Aveyron.

Concerning the ulna (Figure 6c), the first two axes of the PCA explain 28.61% of the total variance. The first axis of the PCA allows us to separate the specimens of *T. aquitania*, represented at the maximum end of the axis, and the specimens of *T. europaea* represented at the minimum end of the axis. *T. occidentalis* is in an intermediate position between the other two species. Indeed, three areas of the bone seem to differ particularly between the two morphologies (Figure 10). The triceps muscle area of insertion is slightly more extended (enlarged surface between the anterior and posterior edge of this area) in *T. aquitania* than in *T. europaea* (Figure 10e). The posterior crest is also slightly more extended posteriorly in its proximal end in *T. aquitania* and is more rounded than in *T. europaea*. Nevertheless, this crest is shorter in its proximo‐distal axis in *T. aquitania* and the ulna curvature starts more proximally (Figure 10c). The distal part of the ulna also seems to differ between the two species (Figure 10d). This difference is related to the ulna curvature which is more pronounced, posteriorly and ventrally, in *T. aquitania*. The distal end of the ulna of these specimens is consequently more antero‐dorsal.

The second axis of the PCA allows us to separate the specimens of *T. aquitania* into two groups corresponding to the geographic origin of the specimens. The MANOVA confirmed that ulnae differ significantly between these two groups (*F* = 18.685, *P* = 3.822e‐4). The maximum of axis 2 mainly explains the shape of the ulnae of the specimens of *T. aquitania* originating from Aveyron, and the minimum mainly explains the shape of the ulnae of the specimens of *T. aquitania* originating from Gironde. Indeed, four areas of the ulna seem to differ particularly (Figure 11). These areas are mainly joint. The radial articular facet is larger and has a less rounded shape in the visualisation corresponding to *T. aquitania* from Gironde (Figure 11c). The semilunar notch, which articulates with the trochlea of the humerus, is, conversely, more pronounced in *T. aquitania* from Aveyron (Figure 11d). The notch is deeper and the anconeus and coronoid processes which border it are slightly more distant on the orange visualisation. The distal end of the ulna also seems to be larger in *T. aquitania* from Aveyron. In these specimens, the cuneiform articular facet is further from the lunar articular facet, and the terminal process is consequently more extended posteriorly (Figure 11e). An area of muscle attachment also seems to differ between the two shapes. The triceps muscle area of insertion offers a slightly extended surface for the triceps muscle attachment in *T. aquitania* from Gironde compared to *T. aquitania* from Aveyron. It is mainly visible on the posterior side of this crest as it seems to extend more posteriorly on the grey shape (Figure 11f). No significant difference between sexes is recorded in the shape of the humerus and ulna (humerus: *F* = 2.060, *P* = 0.305; ulna: *F* = 1.122, *P* = 0.440).

### 3.3 Covariations

The first axis of the PLS (42.92% of the total covariation) is significant (*P* = 0.001; Figure 12). The covariance, therefore, represents 95.73% of the total variance of the humerus and 75.12% of the total variance of the ulna. The rPLS is 0.894, indicating that the humerus and ulna are highly integrated. The first PLS axis is bordered by *T. europaea* (negative values) on one side and *T. aquitania* (positive values) on the other side. *Talpa occidentalis* shows intermediate covariation.

The covariation patterns between the humerus and ulna are nearly the same as the variation patterns highlighted by the visualisations of the shapes associated with the first PCA axis for the humerus and ulna (respectively Figures 7, 10), but involve fewer areas for the humerus. Indeed, the areas concerned are exclusively the lateral lamina, teres tubercle, pectoralis ridge and the lateral epicondyle on the humerus. For the ulna, the parts that covary are all those that change according to the species, that is: the posterior crest, the triceps muscle area of insertion and the bone curvature along with the distal end.

The plot of the second PLS axis between the humerus and ulna (20.56% of the total covariation) is shown in Figure S2. Although it does not represent the main axis of covariation, it does highlight the separation between the two groups of specimens of the species *T. aquitania*. As for the PCA (Figures 6b,c), the Aquitanian moles are grouped according to their locality.

## 4 DISCUSSION

### 4.1 Size and allometry

**FIGURE 5:**
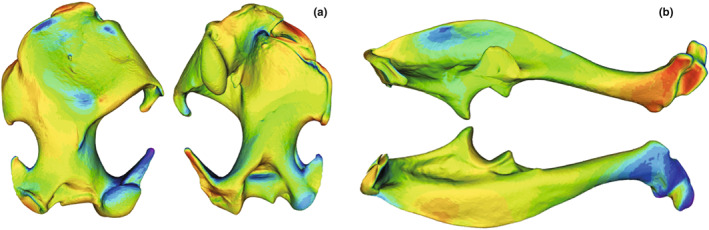
Shape changes of the humerus (a) and ulna (b) between the smallest and largest bones (using the centroid size) are represented: “Cold” colours for areas where the shape of the largest bones recedes within the shape of the smallest bones and “warm” colours where the shape of the largest bones bulges out of the shape of the smallest bones.

Differences in humerus and ulna size exist between the three species. These variations are consistent with the overall size differences of the specimens (Nicolas et al., 2016). Indeed, *T. aquitania*, considered a “giant” species, has the largest humerus and ulna and *T. occidentalis*, considered a “dwarf” species, has the smallest humerus and ulna (Figure 4). However, whatever the size, it always has the same influence on the shape of the bones. There is no effect of species on allometry, which means no evolutionary allometry (Klingenberg, 2016) was detected in our sample (Table S3). In contrast, common static allometry (Klingenberg, 2016) is found for all moles (Table S3). This result is surprising since several studies show that static allometric slopes often vary among mole species, and the different allometric slopes are sometimes taken as an argument to indicate the presence of several separate species (Sansalone et al., 2015). From the visualisations, we can see that the size‐related changes in morphological traits are minimal compared to non‐allometric variations (Figure S3), and they are not identically distributed on the bones (Figure 5).

**FIGURE 6:**
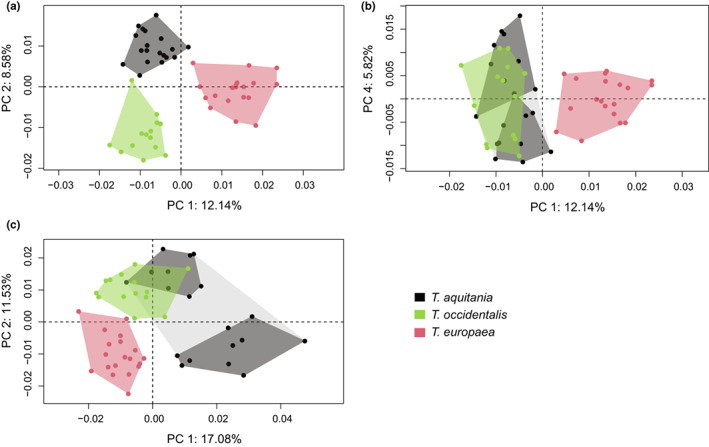
Results of the PCA performed on the morphometric data of the humerus (axes 1–2: a; axes 1–4: b) and ulna (axe 1–2: c). Each individual, represented by a point on the graphs, was coloured according to its species.

**FIGURE 7:**
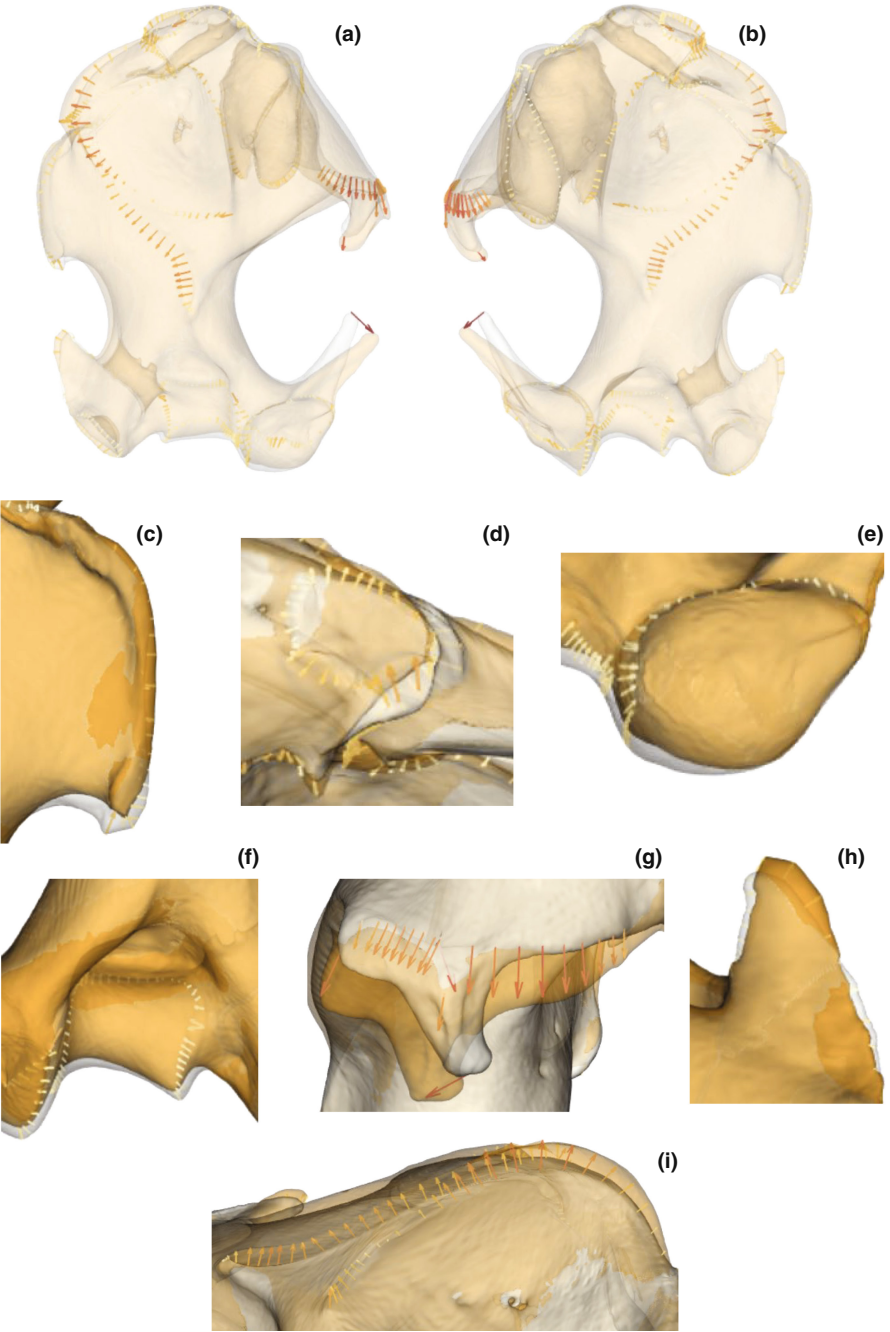
Comparison of the shape of humeri corresponding to the minimum in clear grey (*T. occidentalis* and *T. aquitania*) and the maximum in clear orange (*T. europaea*) of the axis 1 of the PCA. The arrows show the differences, at the landmark locations, between the morphologies representing the minimum and maximum of the axis 1. The intensity of the colour is proportional to the length of the arrows. (a) Anterior view of the humerus. (b) Posterior view of the humerus. (c) Teres tubercle. (d) *Subscapularis* ligament facet. (e) Capitulum. (f) Trochlea. (g) Deltoid process. (h) Medial epicondyle. (i) Pectoralis ridge, deltoid tuberosity and lateral lamina.

**FIGURE 8:**
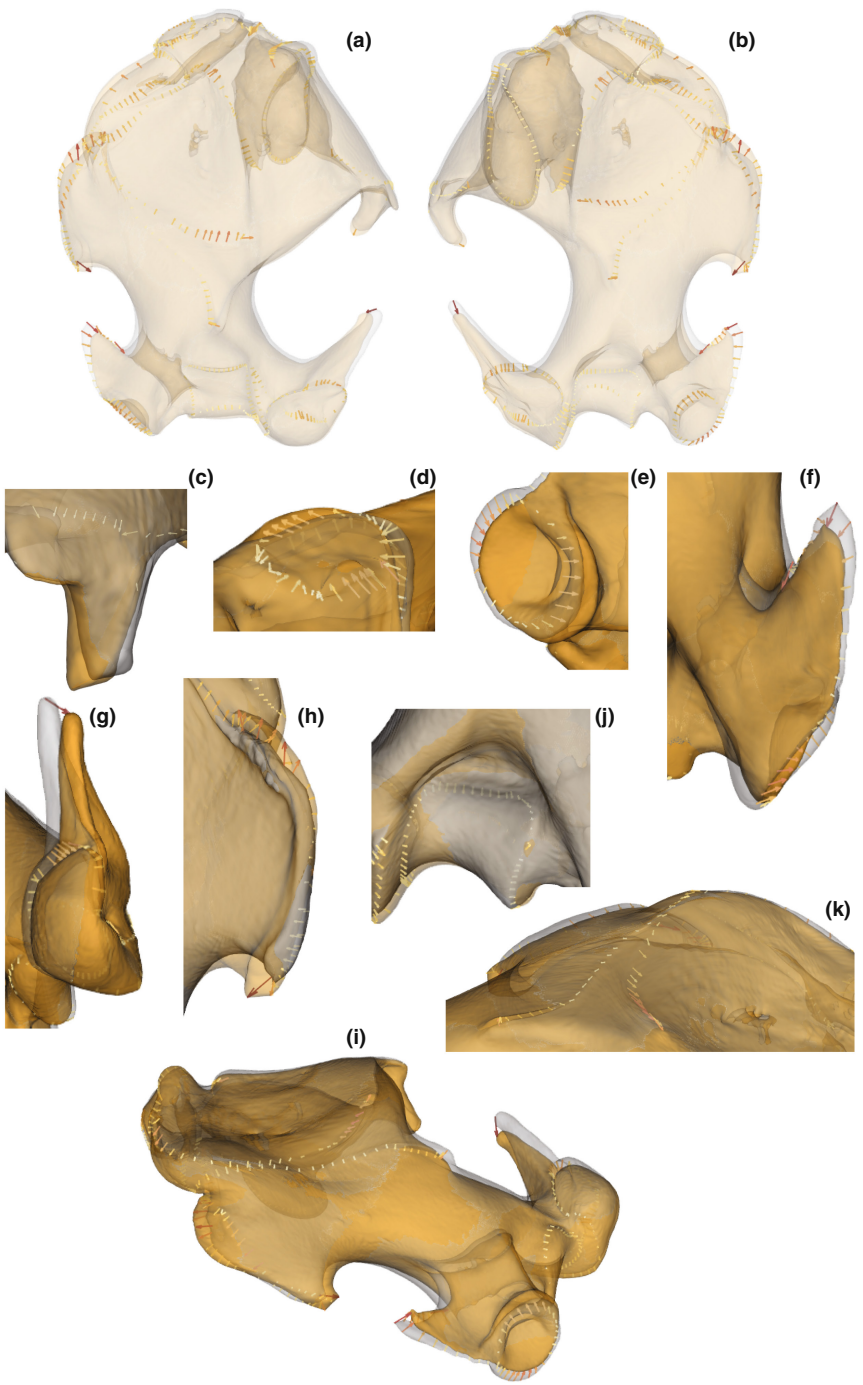
Comparison of the shape of humeri corresponding to the minimum in clear grey (*T. occidentalis*) and the maximum in clear orange (*T. aquitania*) of axis 2 of the PCA. The arrows show the differences, at the landmark locations, between the morphologies representing the minimum and maximum of axis 2. The intensity of the colour is proportional to the length of the arrows. (a) Anterior view of the humerus. (b) Posterior view of the humerus. (c) Deltoid process. (d) *Subscapularis* ligament facet. (e) Distal facet‐like process of the medial epicondyle. (f) Medial epicondyle. (g) Capitulum and lateral epicondyle. (h) Teres tubercle. (i) Antero‐ventral view of the humerus. (j) Olecranon fossa. (k) Pectoralis ridge, deltoid tuberosity and lateral lamina.

**FIGURE 9:**
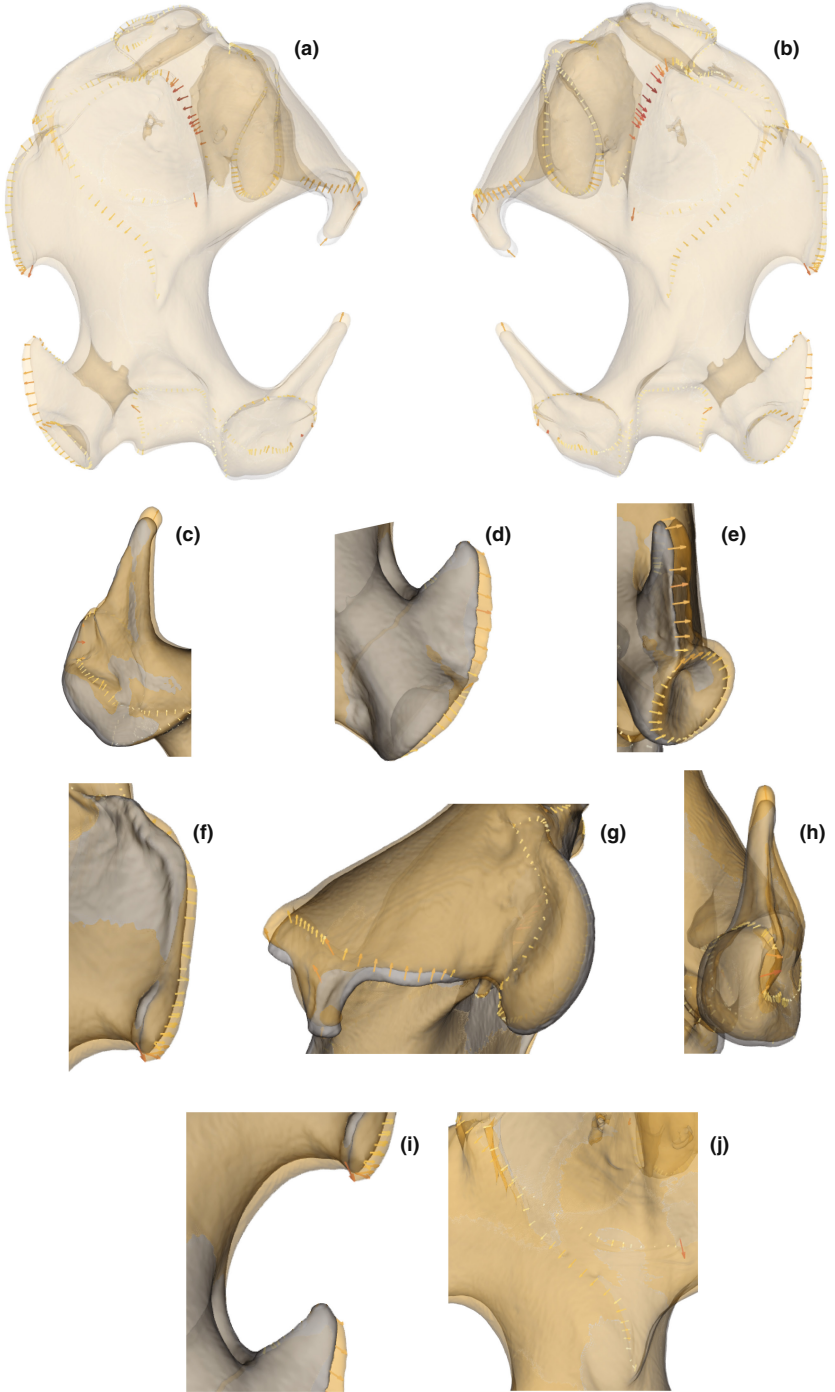
Comparison of the shape of humeri corresponding to the minimum in clear grey (*T. aquitania* of Gironde) and the maximum in clear orange (*T. aquitania* of Aveyron) of axis 4 of the PCA. The arrows show the differences, at the landmark locations, between the morphologies representing the minimum and maximum of axis 4. The intensity of the colour is proportional to the length of the arrows. (a) Anterior view of the humerus. (b) Posterior view of the humerus. (c) Lateral epicondyle. (d) Medial epicondyle. (e) Medial epicondyle and distal facet‐like process of the medial epicondyle. (f) Teres tubercle. (g) Clavicular facet and humeral head. (h) Capitulum and lateral epicondyle. (i) Posterior notch. (j) Pectoralis ridge and deltoid tuberosity.

**FIGURE 10:**
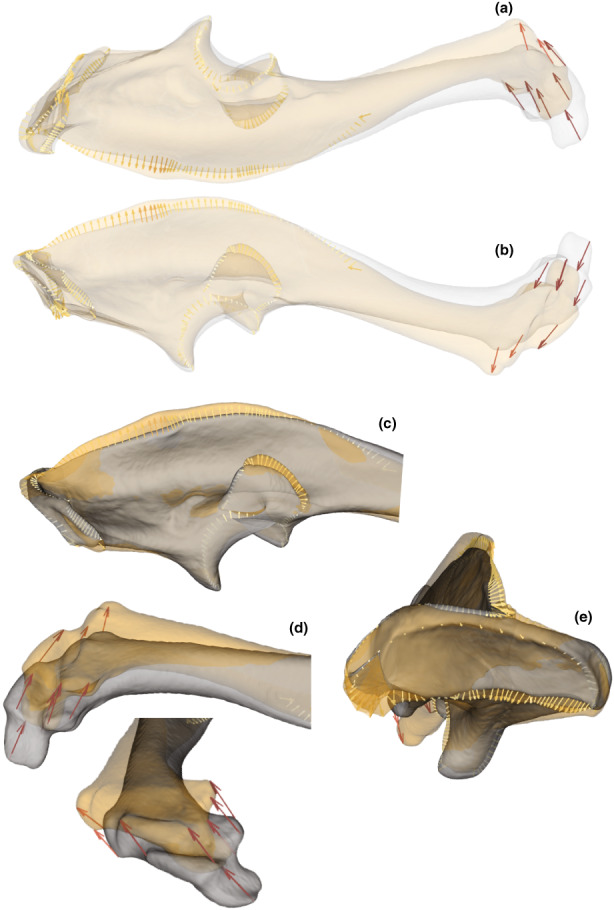
Comparison of the shape of the ulna corresponding to the minimum in clear grey (*T. europaea*) and the maximum in clear orange (*T. aquitania*) of the first axis of the PCA. The arrows show the differences, at the landmark locations, between the morphologies representing the minimum and maximum of axis 1. The intensity of the colour is proportional to the length of the arrows. (a) Ventral view of the ulna. (b) Dorsal view of the ulna. (c) Posterior crest. (d) Distal end. (e) Proximal crest of the olecranon.

**FIGURE 11:**
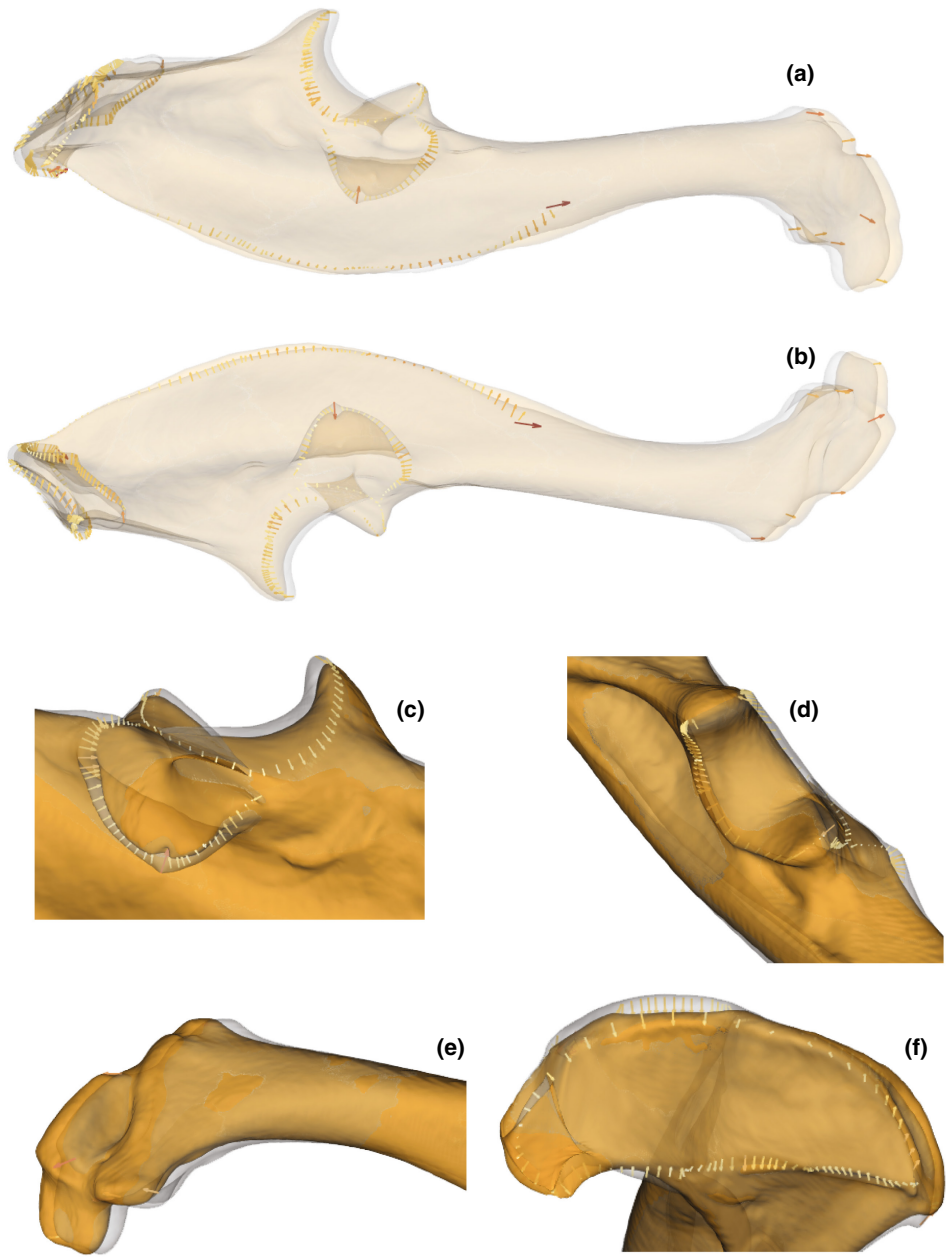
Comparison of the shape of the ulna corresponding to the minimum in clear grey (*T. aquitania* from Gironde) and the maximum in clear orange (*T. aquitania* from Aveyron) of the second axis of the PCA. The arrows show the differences, at the landmark locations, between the morphologies representing the minimum and maximum of axis 2. The intensity of the colour is proportional to the length of the arrows. (a) Ventral view of the ulna. (b) Dorsal view of the ulna. (c) Radial articular facet. (d) Semilunar notch. (e) Distal end. (f) Proximal crest of the olecranon.

**FIGURE 12:**
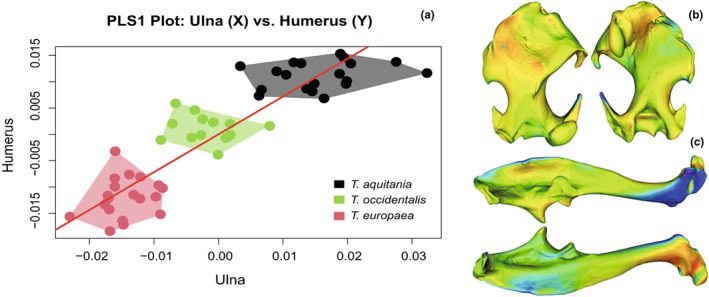
Plot of the first Partial Least Square axis between humerus and ulna (a). The variance of the ulna shape is plotted on the X‐axis and that of the humerus on the Y‐axis. Each individual, represented by a point on the graphs, was coloured according to its species. The red ones belong to *T. europaea*, the green ones belong to *T. occidentalis* and the black ones belong to *T. aquitania*. Humerus (b) and ulna (c) shape changes associated with each extreme of the first axis of the PLS are represented: “Cold” colours for areas where the maximal shape recedes within the minimal shape and “warm” colours where the maximal shape bulges out of the minimal shape.

### 4.2 Interspecific variability

The 3D geometric morphometric analysis allowed us to precisely quantify the extent and nature of the variable regions in the humerus and ulna. The humeral abductor/rotators, elbow extensors, and carpal flexors are important for the production of high force and joint torques during the power stroke (Rose et al., 2013). The application of high out‐force at the manus is realised mainly through the actions of the humeral abductor/rotators (Rose et al., 2013). Specifically, most of the force generated during digging is attributed to the teres major muscle, which acts at the shoulder joint as a humeral abductor muscle and accounts for 75% of total forelimb muscle volume (Gambaryan et al., 2002; Rose et al., 2013). *T. aquitania* has a teres tubercle longer than the other species, which may indicate that it has a stronger or more developed teres major muscle. Thus, this species seems to have a greater digging force than the others. However, the distal facet‐like process of the medial epicondyle, the attachment area of the flexor digitorum profundus muscle, which transmits force distally along the burrowing appendage (Lin et al., 2019) is similar in all three species.

The elbow extensors and palmaris longus must have the functional capacity to enhance lateral out‐force generated by the humeral abductor/rotators (Rose et al., 2013). The attachment areas of triceps brachii muscle (medial and lateral heads), which are involved in the elbow extension, are more developed in *T. europaea* on the humeral side (posterior notch and lateral border of the greater tuberosity, along the intertubercular groove), but they are more developed in *T. aquitania* on the ulnar side (ventral tip, anterior and posterior edges of the proximal side of the olecranon). The interpretation of these variations in bone shape in terms of muscle insertion size and by extension muscle size or strength is not consistent so perhaps there is another function associated with the shape of these parts of the bone. Concerning the anconeus externus muscle, the attachment area is more developed in *T. occidentalis*, on the humerus side. Indeed, the lateral epicondyle is longer. On the ulna side, the attachment area of these muscles corresponds to the posterior crest, which differs in shape with *T. europaea* and *T. occidentalis* having a longer crest proximo‐distally but a less developed one posteriorly. These shape variations could also be associated with differences in digging strategies between the species. Moreover, the medial epicondyle, which is the attachment area of the palmaris longus muscle, used as a carpal flexor and manus broadener, is also longer in *T. occidentalis* than in *T. europaea* or *T. aquitania*. *T. occidentalis* probably has more developed muscles, which produce a greater mechanical force. These muscles are involved in the extension of the forearm at the elbow, the hand and fingers at the wrist. Thus, these abilities are probably more developed in *T. occidentalis*.

Even though muscle attachment regions better reflect ecological differences than articular regions (Sansalone et al., 2022), joints also have an important role in the strength and stability of movement. Concerning the humero‐ulnar joint, *T. occidentalis has* a deeper olecranon fossa and a more developed anterior surface of the trochlea on the humerus side. Consistently, on the ulnar side, the trochlear incision is more marked (deeper, more distant process). During scratching strokes, which are used to tunnel through compact substrates, the ulna is continuously rotated laterally to sweep soil in both lateral and caudal directions (Lin et al., 2019). This motion may be reinforced by the expanded humeral trochlea and the enlarged humeral epicondyles, which prevent elbow dislocation during lateral rotation of the ulna (Gambaryan et al., 2002). The olecranon fossa, in turn, is thought to act as a pivot, allowing the ulna to glide around the expanded humeral trochlea (Lin et al., 2019). Thus, *T. occidentalis* appears to have the capacity to evolve in more compact environments than other species, which is consistent with its higher mechanical strength than other species.

Concerning the joint between the radius with the humerus or ulna, the capitulum (humerus‐radius joint) is more developed in *T. occidentalis* and *T. aquitania* compared to *T. europaea*, whereas the opposite is true for the size of the radial articular surface (ulna‐radius joint). As the two joints are close to each other, there may be spatial constraints on this morphological feature, as this space cannot accommodate two “large” joints. These articular surfaces, by their nature, allow for great strength and security (Edwards, 1937). It is possible that these two patterns reflect two adaptations with the same consequence: ensuring the stability of the elbow joint. Indeed, species can adapt to similar environmental pressures in different ways (Losos, 2011; Wainwright et al., 2005). Many different phenotypes can produce very similar functional outputs, known as many‐to‐one mapping, and divergent morphologies can have convergent functional performance (Alfaro et al., 2005; Losos, 2011; Renaud et al., 2018; Wainwright et al., 2005). Especially in moles, it is likely that natural selection has favoured the evolution of humeral morphologies characterized by high performance (low stress) rather than promoting the optimisation of humeral mobility. However, the trade‐off between humeral strength and mobility has allowed different morphologies to have similar fitness in the subterranean environment (Losos, 2011; Marks & Lechowicz, 2006).

### 4.3 Covariation of humerus and ulna in the three species

The study of the shape of the humerus and ulna highlights an interspecific variability but also shows that the morphological variations of both bones are not independent. Indeed, variation in humeral shape is associated with variation in ulnar shape. Individual analyses of morphological variations in the humerus and ulna revealed similar morphological variability explained by the same parameter: species. These similarities suggested a strong integration between the humerus and ulna, which was confirmed by the study of the covariation of these bones. As expected, the strong ecological constraints induced by the fossorial lifestyle lead to a functional selection on the morphology of these bones. However, the level of morphological covariation between the humerus and ulna is not exactly the same depending on the species. In other words, the anatomical adaptations of the humerus‐ulna functional unit are different according to the species.

Furthermore, the covariation between the humerus and ulna in moles of the genus *Talpa* is dominated by variation in the attachment areas of muscles and particularly of the attachment areas of shoulder muscles concerning the humerus, which affect the mechanical force deployed during the locomotion and digging, as mentioned above. The areas of greatest variation are largely confined to the muscle insertion areas, suggesting a functional origin of this shape covariation.

*T. aquitania* seems to have a different covariation pattern than the other two species. Indeed, in addition to a strong covariation in the shape of the muscle attachment areas between the ulna and humerus in all three species, there is a covariation in the general bone structure and joint shape in *T. aquitania* that varies according to locality.

### 4.4 Intraspecific variability

Identification of all the *T. aquitania* specimens, included in the study, was based both on morphological (fused eyelids, body size, dental characteristics) and molecular (cytochrome b gene) features (Nicolas et al., 2017). Thus, the morphological variation mentioned previously concerning specimens of this species corresponds to intraspecific variations and not to species identification mistakes. Furthermore, molecular variation within the specimens from Gironde and Aveyron of this species already appeared in the results of Nicolas et al. (2017). Phylogenetic analysis of Cytochrome b (a mitochondrial gene) highlighted the presence of 4 sublineages within *T. aquitania*. Two of these sublineages, displaying a divergence time estimated to 0.349 ± 0.050 My, correspond, on the one hand, to all the specimens from Gironde and, on the other hand, to all the specimens from Aveyron included in this study.

However, surprisingly, these intraspecific anatomical changes do not have the same intensity for the humerus and ulna. Indeed, the division into two groups of the *T. aquitania* species, visible on the second axis (14.26%) of the ulna PCA, is not visible on the two first axes of the humerus PCA. This distinction between the Gironde and Aveyron groups in the *T. aquitania* species is only visible on the fourth axis of the humerus PCA, which represents only 6.32% of the variance. This observation could be explained as follows: the humerus and ulna are highly integrated, forming a functional unit. Their shape varies between species to suit their environmental and functional constraints. However, there are also more general bone shape and joint shape changes that occur mostly on the ulna, and to a lesser degree on the humerus, and which are related to the locality of the individuals. The soil properties could explain this difference in intensity between the humerus and ulna. It was found that moles do not show the same burrowing behaviour depending on the compactness of the soil. Indeed, in loose substrates, moles use compacting strokes to move the substrate upwards while scratching strokes are used to advance a tunnel in compact substrates (Lin et al., 2019). However, Lin et al. (2019) showed that distal joint movements differed between behaviours and thus between substrates, whereas movements at the shoulder joint were comparable regardless of substrate. In contrast to the stereotyped movements at the shoulder (action of the teres major muscle at the humerus), elbow movements were observed to differ between compacting and scratching strokes, particularly at the late stage of forelimb retraction. At the end of the retraction phase of the compacting stroke, the ulna resists retraction (i.e., the elbow resists flexion), which aids in compressing the substrates into the wall of the tunnel. This motion reinforces the tunnel wall whilst only involving a single stroke. By contrast, the ulna is continuously rotated laterally during scratching strokes to sweep soil in both lateral and caudal directions (Lin et al., 2019). It could be that the two localities hosting *T. aquitania* do not have the same soil compactness. We know that the shape of the bones is impacted by functional constraints linked to the ecology of the environment (Currey, 2002; Kley & Kearney, 2007). As the humerus is much less affected by soil compactness than the ulna, the shape of the humerus is less affected by locality than the shape of the ulna. 3D visualisations allowed us to identify these morphological variations existing between the ulnae of *T. aquitania* from the Gironde department and *T. aquitania* from the Aveyron department. Most of this variation is located in joint areas. The specimens from Gironde possess a greater articular facet with the radius but the articular surfaces of the carpal bones and the trochlea of the humerus are conversely smaller. A slightly larger attachment area of the triceps muscle is also noticeable in these specimens. As mentioned before, Lin et al. (2019) showed that more compact soils require ulnar lateral rotation while digging in moles, while loose substrates mostly require the elbow to resist flexion. Gambaryan et al. (2002) and Lin et al. (2019) also suggest that elbow extensor function in moles is more to resist flexion at the elbow joint rather than to participate in the elbow extension. The triceps muscle, which possesses a greater attachment area in the Gironde specimens of *T. aquitania*, is an elbow extensor (Freeman, 1886). Furthermore, ulnar lateral rotation requires an expanded humerus trochlea to avoid elbow dislocation. The semilunar notch, which articulates with the humerus trochlea, is more marked in the Aveyron specimens. The specimens originating from Gironde would, thus, possess a greater ability to resist flexion at the elbow joint and less supported ulnar lateral rotation. So, individuals from Aveyron would be better adapted to living in compact soils and individuals from Gironde would be better adapted to living in loose soils.

The influence of locality on the morphology of subterranean mammals has already been shown. For instance, Marcy et al. (2013) showed that pocket gopher species (*Thomomys spp*., Geomyidae), like other subterranean mammals, display an allopatric distribution and morphological variations linked to soil hardness. These authors showed that each species lives where the type of soil reduces the energetic cost of digging depending on its morphology. Further studies are therefore needed to conclude on this intraspecific variation of the ulna in *T. aquitania*, especially pedologic studies of the soils of Aveyron and Gironde where the specimens were captured.

## 5 CONCLUSION

To conclude, as expected, interspecific anatomical differences in the humerus and ulna exist among *T. occidentalis*, *T. aquitania* and *T. europaea*. Shape changes are mostly located at the level of joints and muscle attachments. As the species tend to live in allopatry and the fossorial lifestyle induces strong ecological constraints, interspecific variations could be explained by the properties of the environment in which they live, such as the compactness of the soil. The second investigation of this study was to understand whether functional stresses impacted the humerus and ulna as independent modules or as functional unit. Results show that the humerus and ulna are highly integrated. The covariation between the humerus and ulna in moles of the genus *Talpa* is dominated by variation in the attachment areas and particularly of the attachment areas of shoulder muscles concerning the humerus, which affect the mechanical force deployed during locomotion and digging. This study also highlighted that in the new species, *T. aquitania*, variations in anatomical structure (general shape and joints) existed and were related to the locality of collect of the individuals. However, these changes affected the ulna more than the humerus, as the latter has a much smaller range of motion and is invariant to any burrowing behaviour performed by the moles. Ecological data are needed to place the observed patterns in an evolutionary context, as they allow a better understanding of the selective pressures operating concretely on the locomotion system in different populations. Furthermore, comparing individuals living in allopatric and sympatric areas, and a precise description of the habitat in which they are captured when they are in areas of sympatry (i.e., are they also in syntopy?) would be a good direction in which to continue this research.

## AUTHOR CONTRIBUTIONS

P.C. and E.K. carried out the acquisition of morphological data, performed the statistical analyses, the interpretation of data and drafted the manuscript. These authors have equally contributed to this article. R.C. and V.N. conceived and designed the study, collecting data, statistical analyses and interpreting of data. C.H. contributed to collecting data. A.D. contributed to collecting data and to carried out the acquisition of 3D data. All authors revised and approved the manuscript.

## CONFLICT OF INTEREST

The authors declare that they have no conflict of interest.

## Supporting information


Figure S1

Figure S2

Figure S3

Figure S4

Appendix S1

## Data Availability

The data that support the findings of this study are available from the corresponding author upon reasonable request.
