# Supplementary material for: Comparative morpho‐functional analysis of the humerus and ulna in three Western European moles species of the genus Talpa, including the newly described T. aquitania
Source: J Anat. 2022 Sep 26;242(2):257–76. doi: 10.1111/joa.13772 (PMC9877487; doi:10.1111/joa.13772)
Supplement: Supplementary file 5 — Appendix S1 [file JOA-242-257-s001.docx]

**Supplementary material**

**Table S1** Main information concerning the studied individuals.

| **Collection identifiant** | **Species** | **Sex** | **Locality** | **Humerus** | **Ulna** |
| --- | --- | --- | --- | --- | --- |
| 21550 | *T. occidentalis* | M | Madrid, Spain | Left symmetrized | Left symmetrized |
| 21534 | *T. occidentalis* | M | Madrid, Spain | Left symmetrized | Left symmetrized |
| 21521-21559 | *T. occidentalis* | F | Madrid, Spain | Left symmetrized | Left symmetrized |
| 21521-21559 BIS | *T. occidentalis* | F | Madrid, Spain | Left symmetrized | Left symmetrized |
| 18088-18090 | *T. occidentalis* | / | Lugo, Spain | Left | Left |
| 18088-18090 BIS | *T. occidentalis* | / | Lugo, Spain | Left | Left |
| 18086-18089 | *T. occidentalis* | M | Lugo, Spain | Left | Left |
| 18086-18089 BIS | *T. occidentalis* | M | Lugo, Spain | Left | Left |
| 18074 | *T. occidentalis* | F | Lugo, Spain | Left symmetrized | Left |
| 18082 | *T. occidentalis* | F | Lugo, Spain | Left | Left |
| 18087 | *T. occidentalis* | M | Lugo, Spain | Left | Left |
| 18081 | *T. occidentalis* | M | Lugo, Spain | Left | Left |
| 21548 | *T. occidentalis* | M | Madrid, Spain | Left | Left |
| 21538 | *T. occidentalis* | F | Madrid, Spain | Left | Left |
| MNHN-ZM-2018-0594 | *T. europaea* | M | Plouër-sur-Rance, Côtes d'armor (22), France | Left | Left |
| MNHN-ZM-2018-0598 | *T. europaea* | M | Plouër-sur-Rance, Côtes d'armor (22), France | Left | Left |
| MNHN-ZM-2018-0599 | *T. europaea* | F | Plouër-sur-Rance, Côtes d'armor (22), France | Left | Left |
| MNHN-ZM-2018-0595 | *T. europaea* | F | Plouër-sur-Rance, Côtes d'armor (22), France | Left | Left |
| MNHN-ZM-2018-0603 | *T. europaea* | F | Ploubalay, Côtes d'armor (22), France | Left | Left |
| MNHN-ZM-2018-0604 | *T. europaea* | F | Ploubalay, Côtes d'armor (22), France | Left | Left |
| MNHN-ZM-MO-1993-3218 | *T. europaea* | M | Vaugrigneuse, Essonne (91), France | Left | Left |
| MNHN-ZM-MO-1993-3219 | *T. europaea* | M | Vaugrigneuse, Essonne (91), France | Left | Left |
| MNHN-ZM-MO-1993-3221 | *T. europaea* | F | Vaugrigneuse, Essonne (91), France | Left | Left |
| MNHN-ZM-MO-1993-3222 | *T. europaea* | F | Vaugrigneuse, Essonne (91), France | Left | Left |
| MNHN-ZM-MO-1993-3234 | *T. europaea* | M | Vaugrigneuse, Essonne (91), France | Left symmetrized | Left symmetrized |
| MNHN-ZM-MO-1983-778 | *T. europaea* | F | Vaugrigneuse, Essonne (91), France | Left | Left |
| MNHN-ZM-MO-1993-1708 | *T. europaea* | F | Vaugrigneuse, Essonne (91), France | Left symmetrized | Left symmetrized |
| MNHN-ZM-MO-1993-3220 | *T. europaea* | M | Vaugrigneuse, Essonne (91), France | Left | Left |
| MNHN-ZM-MO-1993-3235 | *T. europaea* | F | Vaugrigneuse, Essonne (91), France | Left | Left |
| MNHN-ZM-2018-0600 | *T. europaea* | F | Pleurtuit, Ille-et-Vilaine (35), France | Left | Left |
| MNHN-ZM-2018-0596 | *T. europaea* | F | Saint-Briac, Ille-et-Vilaine (35), France | Left | Left |
| MNHN-ZM-2018-0605 | *T. europaea* | F | Saint-Lunaire, Ille-et-Vilaine (35), France | Left | Left |
| MNHN-ZM-2018-0606 | *T. europaea* | F | Saint-Lunaire, Ille-et-Vilaine (35), France | Left | Left |
| MNHN-ZM-2018-2246 | *T. aquitania* | F | Cantoin, Aveyron (12), France | Left | Left |
| MNHN-ZM-2018-2245 | *T. aquitania* | F | Cantoin, Aveyron (12), France | Left | Left |
| MNHN-ZM-2018-2240 | *T. aquitania* | M | Cantoin, Aveyron (12), France | Left | Left |
| MNHN-ZM-2018-2248 | *T. aquitania* | M | Cantoin, Aveyron (12), France | Left | Left |
| MNHN-ZM-2018-2243 | *T. aquitania* | F | Cantoin, Aveyron (12), France | Left symmetrized | Left symmetrized |
| MNHN-ZM-2018-2241 | *T. aquitania* | M | Cantoin, Aveyron (12), France | Left | Left |
| MNHN-ZM-2018-2244 | *T. aquitania* | M | Cantoin, Aveyron (12), France | Left | Left |
| MNHN-ZM-2018-2239 | *T. aquitania* | F | Cantoin, Aveyron (12), France | Left | Left |
| MNHN-ZM-2018-2242 | *T. aquitania* | F | Cantoin, Aveyron (12), France | Left | Left |
| MNHN-ZM-2017-2254 | *T. aquitania* | M | Izon, Gironde (33), France | Left | Left |
| MNHN-ZM-2017-2257 | *T. aquitania* | M | Landiras, Gironde (33), France | Left | Left |
| MNHN-ZM-2017-2258 | *T. aquitania* | F | Landiras, Gironde (33), France | Left | Left |
| MNHN-ZM-2017-2259 | *T. aquitania* | M | Landiras, Gironde (33), France | Left | Left |
| MNHN-ZM-2017-2260 | *T. aquitania* | F | Landiras, Gironde (33), France | Left | Left |
| MNHN-ZM-2017-2261 | *T. aquitania* | M | Cestas, Gironde (33), France | Left | Left |
| MNHN-ZM-2017-2271 | *T. aquitania* | M | Saint-Sulpice-et-Cameyrac, Gironde (33), France | Left | Left |
| MNHN-ZM-2017-2273 | *T. aquitania* | F | Landiras, Gironde (33), France | Left | Left |
| MNHN-ZM-2017-2274 | *T. aquitania* | F | Landiras, Gironde (33), France | Left | Left |
| MNHN-ZM-2017-2275 | *T. aquitania* | F | Landiras, Gironde (33), France | Left | Left |

**Table S2** Location and definitions of anatomical landmarks and curves used for the humerus (a) and ulna (b) templates. Each curve is bounded by two anatomical landmarks.

| **Anatomical landmark** | **Anatomical location** | | **Curve (defined anatomical structure)** | **Area type** |
| --- | --- | --- | --- | --- |
| 1. **Humerus** | | | | |
| 1. | | Most dorsal point of the posterior part of the attachment of brachialis muscle | Attachment area of triceps brachii | Attachment area of the medial head of the triceps brachii |
| 26. | | Postero-ventral tip of the lesser tuberosity |  |  |
| 16. | | Most distal point of the teres tubercle | Teres tubercle | Attachment area of the teres major |
| 17. | | Most proximal point of the teres tubercle |  |  |
| 13. | | Most ventral point of the distal facet-like process of the medial epicondyle | Medial epicondyle | Attachment areas of the pronator teres, palmaris longus, anconeus internus |
| 14. | | Most proximal tip of the medial epicondyle |  |  |
| 15. | | Most distal point of the posterior notch |  |  |
| 20. | | Most anterior point of the deltoid tuberosity | Lateral lamina and pectoralis ridge  then  medial lamina | Attachment areas of the pectoralis major (the anterior long, posterior abdominal and posterior deep pectoralis) and clavicular head of the deltoid |
| 22. | | Most postero-ventral point of the clavicular facet |  |  |
| 23. | | Most proximal tip of the medial lamina on lesser tuberosity |  |  |
| 24. | | The intersection point between the lateral lamina and the medial lamina |  |  |
| 1. | | - | Deltoid process | Attachment areas of the lateral head of the triceps brachii and brachialis |
| 5. | | Tip of the deltoid process |  |  |
| 6. | | Most posterior intersection point between the deltoid process and the clavicular facet |  |  |
| 7. | | Most anterior intersection point between the deltoid process and the clavicular facet |  |  |
| 5. | | - | Deltoid process | Attachment area of the scapular head of the deltoid |
| 24. | | - | Subscapularis ligament facet | Attachment area of the subscapularis |
| 25. | | Most postero-distal intersection point between the lesser tuberosity and the lateral lamina |  |  |
| 27. | | Most ventral point of the subscapularis ligament facet |  |  |
| 8. | | Most proximal tip of the lateral epicondyle | Lateral epicondyle | Attachment area of the anconeus externus, extensor carpi radialis, extensor digitorum communis |
| 13. | | - | Distal facet-like process of the medial epicondyle | Attachment area of the flexor digitorum profundus |
| 28. | | Most anterior point of the pectoralis ridge | Pectoralis ridge | Attachment areas of the pectoralis major and biceps support |
| 29. | | Point of the maximum concavity of the proximal part of the intertubercular groove |  |  |
| 17. | | - |  |  |
| 18. | | Point of the maximum concavity of the posterior part of the intertubercular groove |  |  |
| 19. | | The intersection point between the lateral lamina and the bicipital notch |  |  |
| 2. | | Most posterior point of the clavicular facet | Clavicular facet | Clavicle joint |
| 3. | | Most postero-dorsal point of the clavicular facet |  |  |
| 4. | | Most antero-dorsal point of the clavicular facet |  |  |
| 21. | | Most antero-ventral point of the clavicular facet |  |  |
| 22. | | - |  |  |
| 0. | | Most proximal point of the humeral head | Humeral head | Scapula joint |
| 10. | | Point of the maximum convexity of the anterior part of the trochlea | Trochlea | Ulna joint |
| 11. | | Most distal point of the trochlea |  |  |
| 12. | | Point of the maximum concavity of the ventral part of the olecranon fossa |  |  |
| 9. | | Most postero-proximal point of the capitulum | Capitulum | Radius joint |
| 1. **Ulna** | | | | |
| 0. | | Most antero-ventral point of the anconeus process | Ventral edge of the  semilunar notch | Articulation with the  humerus trochlea |
| 2. | | Most antero-dorsal point of the coronoid process |  |  |
| 2. | | - | Dorsal edge of the  semilunar notch |  |
| 1. | | Most antero-dorsal point of the anconeus process |  |  |
| 3. | | Proximal point of contact between the semilunar notch and the radial articular facet | External outline of the radial articular facet | Articulation with the  proximal part of the radius |
| 4. | | Distal point of contact between the semilunar notch and the radial articular facet |  |  |
| 5. | | Point of contact between the posterior crest and the proximal crest of the olecranon | Posterior edge of the  posterior crest | Attachment areas of the anconeus externus, abductor pollicis longus, extensor indicis et pollicis longus, flexor digitorum profondus, flexor carpi ulnaris |
| 6. | | Most distal point of the posterior crest |  |  |
| 7. | | Ventral tip of the proximal crest of the olecranon | Posterior edge of the  triceps area of insertion | Attachment areas of the triceps |
| 8. | | Most proximo-dorsal point of the proximal crest of the olecranon |  |  |
| 7. | | - | Anterior edge of the  triceps area of insertion |  |
| 8. | | - |  |  |
| 9. | | Dorsal tip of the proximal crest of the olecranon | Distal edge of the dorsal part of the proximal crest | Attachment areas of the anconeus externus, extensor digiti minimi, extensor indicis et pollicis longus |
| 5. | | - |  |  |
| 5. | | - | Distal edge of  the ventral part of the proximal crest | Attachment areas of the anconeus internus, flexor carpi ulnaris |
| 10. | | Tip of the postero-ventral hook of the proximal crest of the olecranon |  |  |
| 10. | | - |  |  |
| 7. | | - |  |  |
| 11. | | Most distal tip of the terminal process | - | Articulation with the cuneiform (carpal bone) |
| 12. | | Most anterior point of the cuneiform articular facet | - |  |
| 13. | | Point of contact and point of maximum curvature between the styloid process and the cuneiform articular facet | - |  |
| 18. | | Most postero-proximal point of the cuneiform articular facet | - |  |
| 14. | | Most proximal tip of the styloid process | - |  |
| 15. | | Most distal tip of the styloid process | - |  |
| 16. | | Most antero-proximal point of the lunar articular facet | - | Articulation with the distal part of the radius |
| 17. | | Most distal point of the lunar articular facet | - |  |

**Table S3** Procrustes ANOVA with species and centroid size for the humerus (a) and ulna (b). Df: Degree of freedom, SS: Sums of squares, MS: Mean of sums of squares, Rsq: R square, Z: Effect-sizes (Z scores).

|  | Df | SS | MS | Rsq | F | Z | Pr(>F) |
| --- | --- | --- | --- | --- | --- | --- | --- |
| 1. **Humerus** | | | | | | | |
| Centroid size | 1 | 0.003085 | 0.0030846 | 0.05784 | 3.4861 | 4.6318 | 0.001 |
| Species | 2 | 0.007591 | 0.0037956 | 0.14235 | 4.2896 | 7.4588 | 0.001 |
| Centroid size:Species | 2 | 0.001948 | 0.0009740 | 0.03653 | 1.1008 | 0.6991 | 0.249 |
| Residuals | 46 | 0.040703 | 0.0008848 | 0.76327 |  |  |  |
| Total | 51 | 0.053327 |  |  |  |  |  |
| **(b) Ulna** | | | | | | | |
| Centroid size | 1 | 0.006595 | 0.0065949 | 0.09704 | 6.0984 | 4.7674 | 0.001 |
| Species | 2 | 0.009168 | 0.0045842 | 0.13491 | 4.2391 | 5.6372 | 0.001 |
| Centroid size:Species | 2 | 0.002453 | 0.0012263 | 0.03609 | 1.1339 | 0.6863 | 0.244 |
| Residuals | 46 | 0.049745 | 0.0010814 | 0.73197 |  |  |  |
| Total | 51 | 0.067961 |  |  |  |  |  |

**Table S4** Pairwise species comparisons according to the centroid size using t test, for the humerus and ulna.

|  | p-value | |
| --- | --- | --- |
|  | **Humerus** | **Ulna** |
| *europaea-aquitania* | 4.1e-05 | 4.0e-06 |
| *europaea-occidentalis* | 1.9e-06 | 2.4e-06 |
| *occidentalis-aquitania* | 1.3e-12 | 2.1e-13 |
